# Malpractice in Midwifery

**Published:** 1846-12

**Authors:** 


9. *Malpractice in Midwifery. Alleged Death from Hæmorrhage.—Charge of Manslaughter against the Medical Attendant.*—An inquest was held at Towcester, on Sát. Aug. 15th last, to inquire into the circumstances attending the death of Ann Smith, who died shortly after her confinement, from the alleged improper treatment and neglect of her medical attendant.

It appeared from the evidence of the witnesses, that the woman was taken in labour of her first child about the middle of the day on Friday. Mr. G—— (M.R.C.S.E.) was sent for, and arrived in about an hour; he told them she was going on well, and that he would return in a short time. He did so in about three quarters of an hour, and remained within call, (but not in the patient's room.) He was several times requested to go up "to help the woman," but did not until immediately before the birth of the child, which took place about six o'clock. He tied and cut the cord in the usual manner, and shortly afterwards, in making traction, broke it off at its attachment to the placenta; a gush of blood followed, but the nurse could not speak with confidence as to the quantity. Mr. G. then gave the patient a dose of tincture of opium, telling the friends there was something more to come, and left, promising to return between eight and nine o'clock. A short time after Mr. G. left, the patient became very faint, and one of the women noticed a pool of blood on the floor under the bed. A messenger was dispatched for Mr. Collier, surgeon, who attended without delay, and found the patient exsanguineous, and in a state of syncope. He immediately removed the placenta, and ordered wine and brandy to be administered. The hæmorrhage ceased after the extraction of the placenta. Mr. Collier then sent home for ammonia and other stimulants; but death took place before the return of the messenger.

In answer to questions from the coroner and some of the jurymen, Mr. Collier said, that he considered it very improper for a medical man to leave a patient while the after-birth was retained, and that, on breaking the cord, he would directly have proceeded to extract the after-birth. The administration of the laudanum at that period was decidedly bad practice, the tendency of its operation being to prevent further contractions of the uterus, which might have effected the expulsion of the placenta naturally. From the cord being torn off at its attachment to the placenta, he believed that an improper degree of force had been employed in the traction.

The coroner then said that "the jury had heard the evidence, which he thought he need not repeat, and it was now their duty to say what was the cause of death, and to decide whether any or what amount of culpability attached to the medical attendant, Mr. G."

In about half an hour the jury returned the following verdict:—"That the cause of the death of Ann Smith was excessive hæmorrhage, and that occasioned principally by the neglect and improper treatment of her medical attendant, Mr. William G."

The coroner said that this did not amount to manslaughter, and consequently he had no authority for proceeding further.

On the following Monday, the husband of the deceased entered a charge of manslaughter against Mr. G. at the Towcester Police Court.

The superintendent of police took Mr. G. into custody, and on Tuesday brought him before the sitting magistrates. The evidence was nearly the same as at the inquest.

The legal adviser of the accused commented strongly on the fact of there being no hæmorrhage, and the woman appearing very comfortable at the time Mr. G. left her. He went on to suggest the probability that the woman's life might have been saved had the women been attentive to the case, and sent for the surgeon at the time the hæmorrhage commenced, instead of waiting till its existence was accidentally discovered by one of them seeing the blood on the floor. It had been proved that Mr. G. had promised to return between eight and nine o'clock, and it was a well-known fact, that formerly medical men were constantly in the habit of leaving their patients for some hours when the after-birth was retained, waiting for the powers of nature to accomplish the delivery, and he believed that it was frequently done now.

After consulting together for a quarter of an hour, the magistrates requested Mr. R. Watkins, surgeon, to give an opinion on the case. Mr. Watkins, having been sworn, confirmed Mr. Collier's view of the case, and detailed the usual mode of treatment under like circumstances. He had never left a midwifery patient till after the removal of the placenta: it was not usual, and he would not do it under any circumstances. If his attendance were required at a second midwifery case, he would not leave the first until the placenta was removed, even though no hæmorrhage had taken place. He was aware that, formerly, in many cases the patient was left before the after-birth came away; he had read of a fatal case, which occurred in the practice of an eminent physician from this cause.

After a lengthened consultation, the magistrates decided, that, although great neglect and improper treatment had been proved, the evidence was not sufficient to support a charge of manslaughter. The case was accordingly dismissed.—*Lancet*, in *Bull. of Med. Science*.

